# Supplementary material for: Feedback mechanisms stabilise degraded turf algal systems at a CO2 seep site
Source: Commun Biol. 2021 Feb 16;4:219. doi: 10.1038/s42003-021-01712-2 (PMC7901039; doi:10.1038/s42003-021-01712-2)
Supplement: Supplementary file 2 — Supplementary Information [file 42003_2021_1712_MOESM2_ESM.pdf]

Supplementary Information

Title: Feedback mechanisms stabilise degraded turf algal systems at a CO_2 seep site

Running Title: Feedback loops lock turf systems in place

Ben P. Harvey[1,*], Ro Allen[2,3*], Sylvain Agostini[1], Linn J. Hoffmann[2], Koetsu Kon[1], Tina C. Summerfield[2], Shigeki Wada[1] and Jason M. Hall-Spencer[1,4]

[1] Shimoda Marine Research Center, University of Tsukuba, 5-10-1 Shimoda, Shizuoka 415-0025, Japan

[2] Department of Botany, University of Otago, Dunedin, New Zealand

[3] The Marine Biological Association, Plymouth, Devon, PL1 2PB, UK

[4] Marine Biology and Ecology Research Centre, University of Plymouth, Plymouth PL4 8AA, UK.

[*] These authors contributed equally.

Correspondence: Ben P Harvey; Email: ben.harvey@shimoda.tsukuba.ac.jp, Tel: (+81) 0558-22-6697

Keywords: Regime shifts, feedback loops, hysteresis, ocean acidification, CO_2 seeps, turf algae

Paper Type: Article

Supplementary Methods

Shikine Island (34°19′9″ N, 139° 12′18″ E) is part of a group of offshore volcanic islands in Japan, termed the Izu Islands. The study area in the reference pCO_2 areas is typical of the region (Agostini et al., 2018) and comprised of a rocky reef habitat with a mixture of both canopy-forming fleshy macroalgae and zooxanthellate scleractinian corals. This balance of both macroalgae and corals is because Shikine Island is located within the subtropical–temperate transition zone. A gradient of seawater CO_2 concentration is present in the area due to the CO_2 seep, and we typically use five sites along the pCO_2 gradient: 'Reference' (mean pCO_2: 410 ± 73), which was outside the influence of the CO_2 seep; 'RCP 2.6′ (mean pCO_2: 493 ± 158); 'RCP 4.5′ (mean pCO_2: 765 ± 159); 'RCP 8.5′ (mean pCO_2: 971 ± 258); and '>RCP 8.5′ (mean pCO_2: 1803 ± 1287, Table S1). The sites were termed 'RCP 2.6′, 'RCP 4.5′, and 'RCP 8.5′ in reference to their equivalent Intergovernmental Panel on Climate Change Representative Concentration Pathway (RCP) scenarios (IPCC, 2013). The >RCP 8.5 site highlights the ecological impacts of ocean acidification beyond predicted levels due to human CO_2 emissions. The elevated pCO_2 areas are not confounded by differences in temperature, dissolved oxygen, total alkalinity or depth relative to reference sites used for comparison (Agostini et al., 2015, 2018). The pCO_2 and temperature conditions are also temporally stable over the long-term while still following natural diurnal variation (Figure S1), as based on month-long measurements taken from Agostini et al. (2018) using durafet pH sensors (Seafet, Sea-Bird Scientific, Canada) .

Supplementary Table 1. Carbonate chemistry of the reference, RCP 2.6, RCP 4.5, RCP 8.5, and >RCP 8.5 sites at Shikine Island, Japan. Modified from Harvey et al. 2019

Station	pH_T	Temp (°C)	Salinity (psu)	A_T (µmol kg^{-1})	pCO_2 (µatm)	DIC (µmol kg^{-1})	HCO_3^- (µmol kg^{-1})	CO_3^{2-} (µmol kg^{-1})	Ωcalcite	Ωaragonite
**Reference**	8.041	23.086	34.129	2281.9	409.965	2007.341	1798.117	196.978	4.76	3.115
	0.067	0.603	0.741	6.80	73.383	38.944	61.612	24.859	0.596	0.392
**RCP 2.6**	7.983	21.437	35.056	2282.93	493.011	2044.255	1855.972	173.103	4.144	2.703
	0.119	1.273	0.125	6.57	158.004	53	81.439	32.771	0.781	0.501
**RCP 4.5**	7.809	22.701	34.455	2283.32	765.545	2122.447	1973.165	126.296	3.043	1.99
	0.075	0.919	0.132	18.53	158.892	27.476	38.887	15.755	0.378	0.244
**RCP 8.5**	7.719	22.896	34.91	2271.84	970.706	2144.537	2008.7	106.928	2.568	1.681
	0.095	0.937	0.211	3.03	257.68	33.169	43.845	17.716	0.423	0.274
**>RCP 8.5**	7.529	22.072	34.723	2277.62	1803.047	2218.975	2088.23	75.92	1.823	1.19
	0.234	1.212	0.742	20.50	1287.448	82.982	82.43	33.368	0.799	0.519

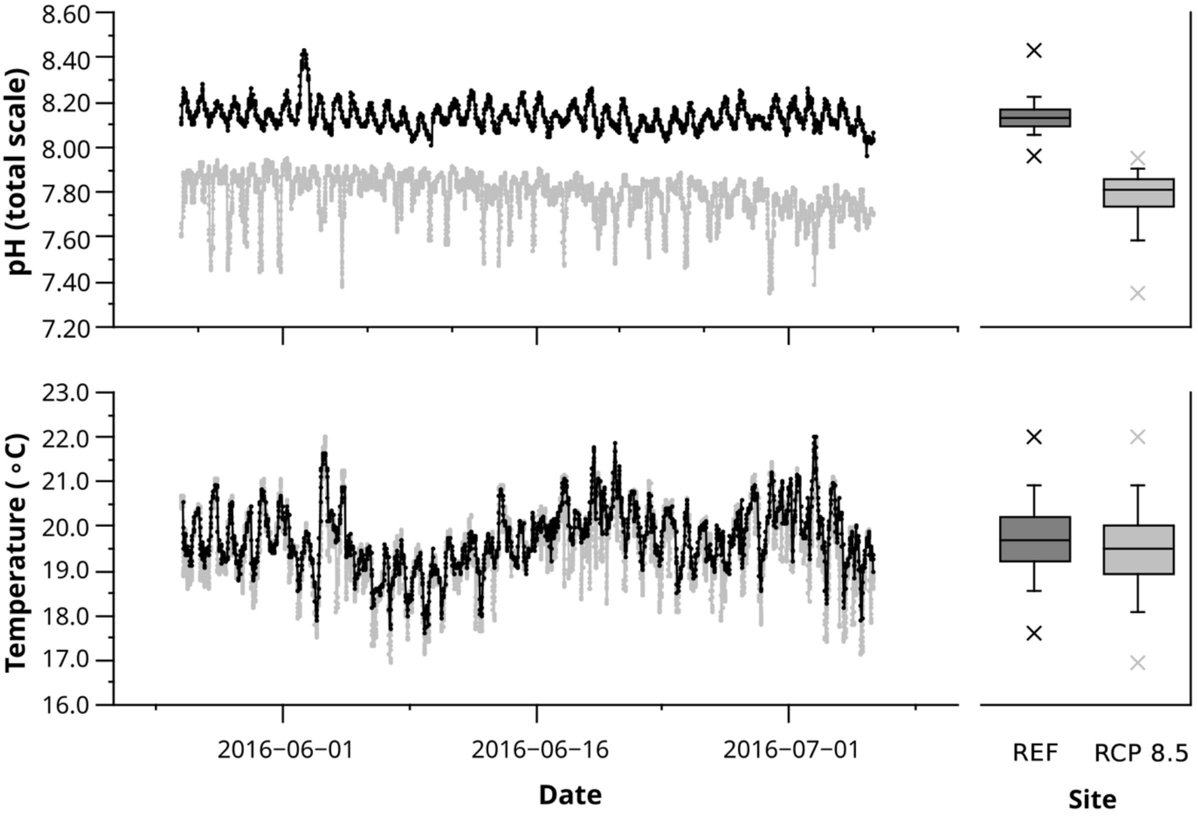

Supplementary Figure 1. Variation of temperature and pH (total scale) over the month of June 2016 at a subtidal control site (REF) and a subtidal elevated CO_2 site (RCP 8.5). Measurements were carried out with SeaFET sensors deployed just above the seafloor. For the boxplots, the line inside indicates the median, the upper and lower hinges correspond to the interquartile range, and the upper and lower whiskers extend to the highest and lowest values that are within 1.5 * the interquartile range of the hinge. Outliers beyond these whiskers are indicated with a cross. Taken from Agostini et al. 2018.

The reference pCO$_2$ site in Shikine Island includes a mix of corals and macroalgae. The coral are predominantly large tabular Acroporids, and sub-massive and encrusting corals (e.g. Favids and Porites) (Agostini et al. 2018). The macroalgae includes a diverse range of taxa, with abundant crustose coralline algae (e.g. Lithophyllum spp.), non-calcareous encrusting algae (e.g. Peyssonneliaceae spp.), and a range of abundant fleshy algae (e.g. *Chondracanthus tenellus, Codium coactum, Gelidium elegans, and Sarcodia ceylanica*) (Agostini et al. 2018). Compared to the reference pCO$_2$ site, the elevated pCO$_2$ locations in Shikine Island greatly differ in terms of their community composition, and are predominantly characterised by extensive expanses of turf algae that dominate the community at the expense of the corals and larger macroalgae (Agostini et al., 2018; Harvey et al., 2019). This results in an ecosystem shift

from complex calcified biogenic habitats towards less complex non-calcified habitats. The turf algae dominating the elevated pCO_2 areas is the diatom *Biddulphia biddulphiana* (J.E. Smith) Boyer, which became increasingly more abundant as CO_2 levels increased due to the CO_2-driven stimulation of its growth and photosynthetic efficiency (Harvey et al., 2019). The turf algae is a tightly packed matrix-like mat of algal filaments (~5-10 cm in height) that extensively covers the substratum, trapping a thick layer of sediment (predominantly sand, ~3-5 cm deep). This thick turf-like algal bloom is capable of supporting an abundant mobile faunal community, although the supported community differs from a typical seaweed community found in the reference pCO_2 conditions.

Supplementary Table 2. The relationship between the percentage cover and measured pH using a negative binomial generalised linear model.

	Estimate	Std. Error	z value	Pr(>|z|)	
**Turf Algae**					
(Intercept)	168.491	31.940	5.275	$1.33e^{-7}$	***
pH_{NBS}	-21.159	4.019	-5.265	$1.40e^{-7}$	***
**Macroalgae**					
(Intercept)	-138.992	23.786	-5.843	$5.12e^{-9}$	***
pH_{NBS}	17.380	2.977	5.838	$5.28e^{-9}$	***
**Corals**					
(Intercept)	-276.04	384.55	-0.718	0.473	n.s.
pH_{NBS}	33.45	47.10	0.710	0.478	n.s.

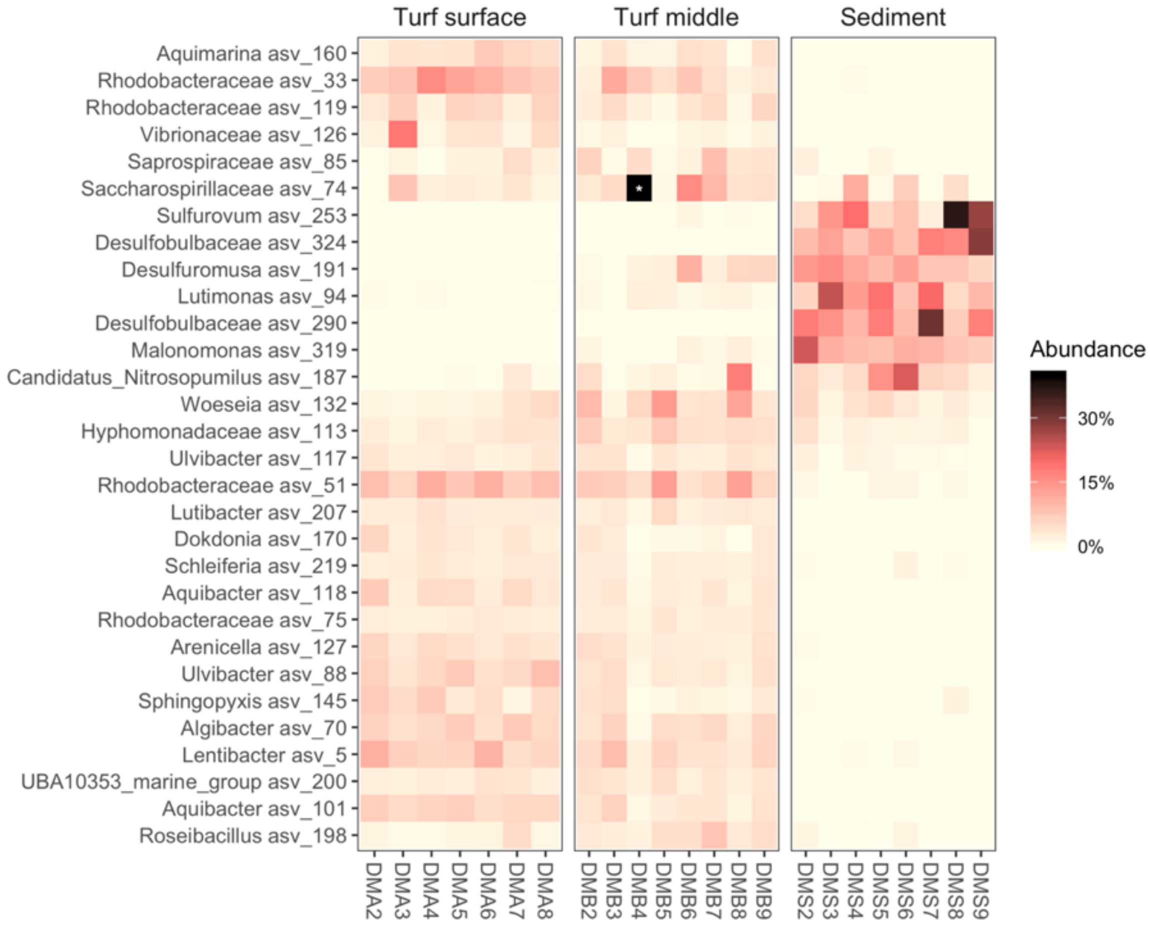

Supplementary Figure 2. Heatmap of microbial community composition at the ASV level, based on Bray-Curtis dissimilarity. The 30 most abundant taxa (based on total reads summed across all samples) are displayed. Taxa are labelled according to the highest available taxonomic classification. Taxa are ordered to represent similarity in distribution across samples, according to the neatmap algorithm (Rajaram & Oono, 2010). Colour bar indicates the relative abundance of 16S rRNA gene reads attributed to an ASV in each sample. The white Asterix (*) indicates a relative abundance of 47% in sample DMB4, which is outside the range of the colour bar.

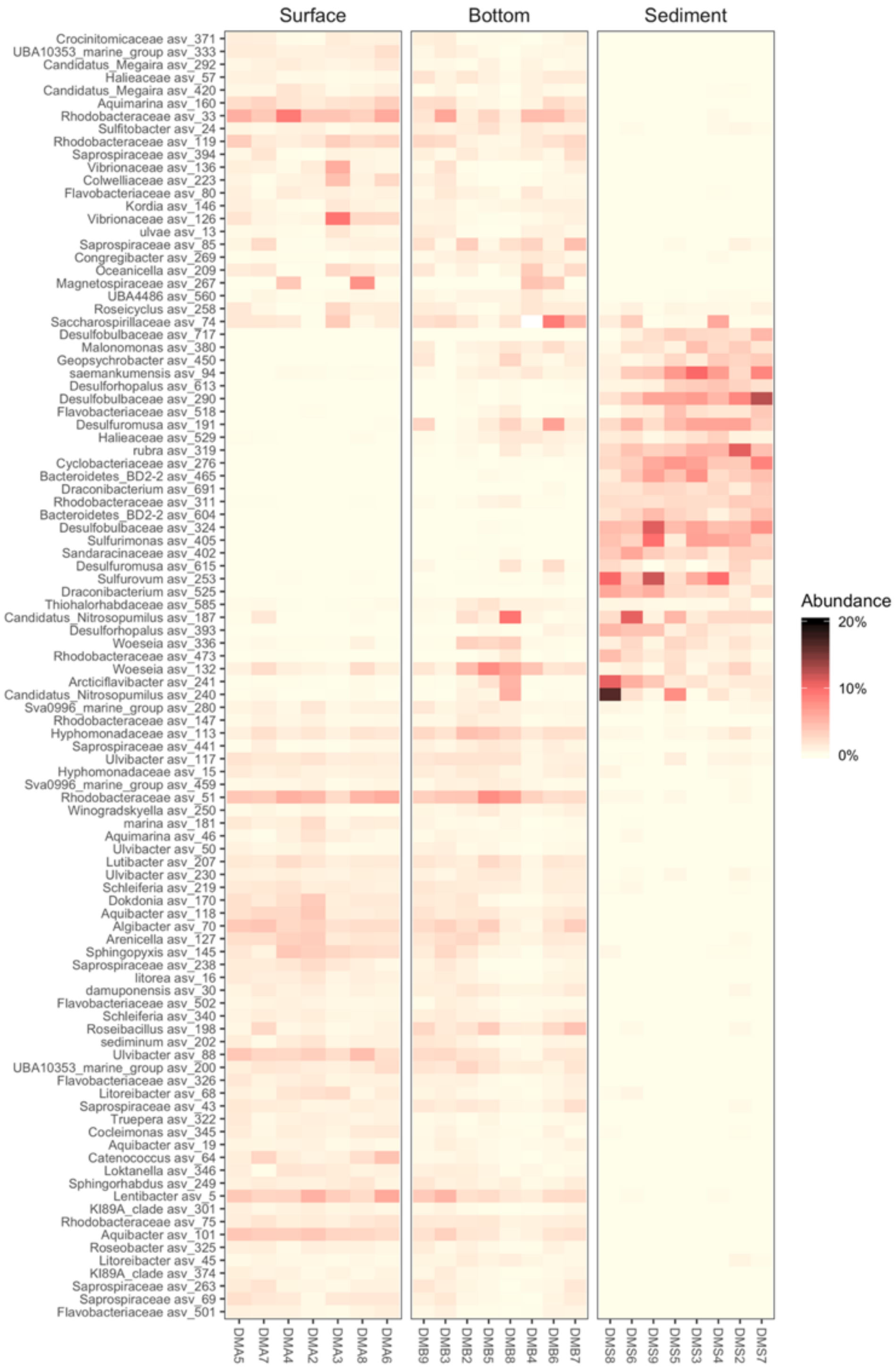

Supplementary Figure 3. Heatmap of microbial community composition at the ASV level, based on Bray-Curtis dissimilarity. Taxa are labelled according to the highest available taxonomic classification. Taxa are ordered to represent similarity in distribution across samples, according to the neatmap algorithm (Rajaram & Oono, 2010). Colour bar indicates the relative abundance of 16S rRNA gene reads attributed to an ASV in each sample.

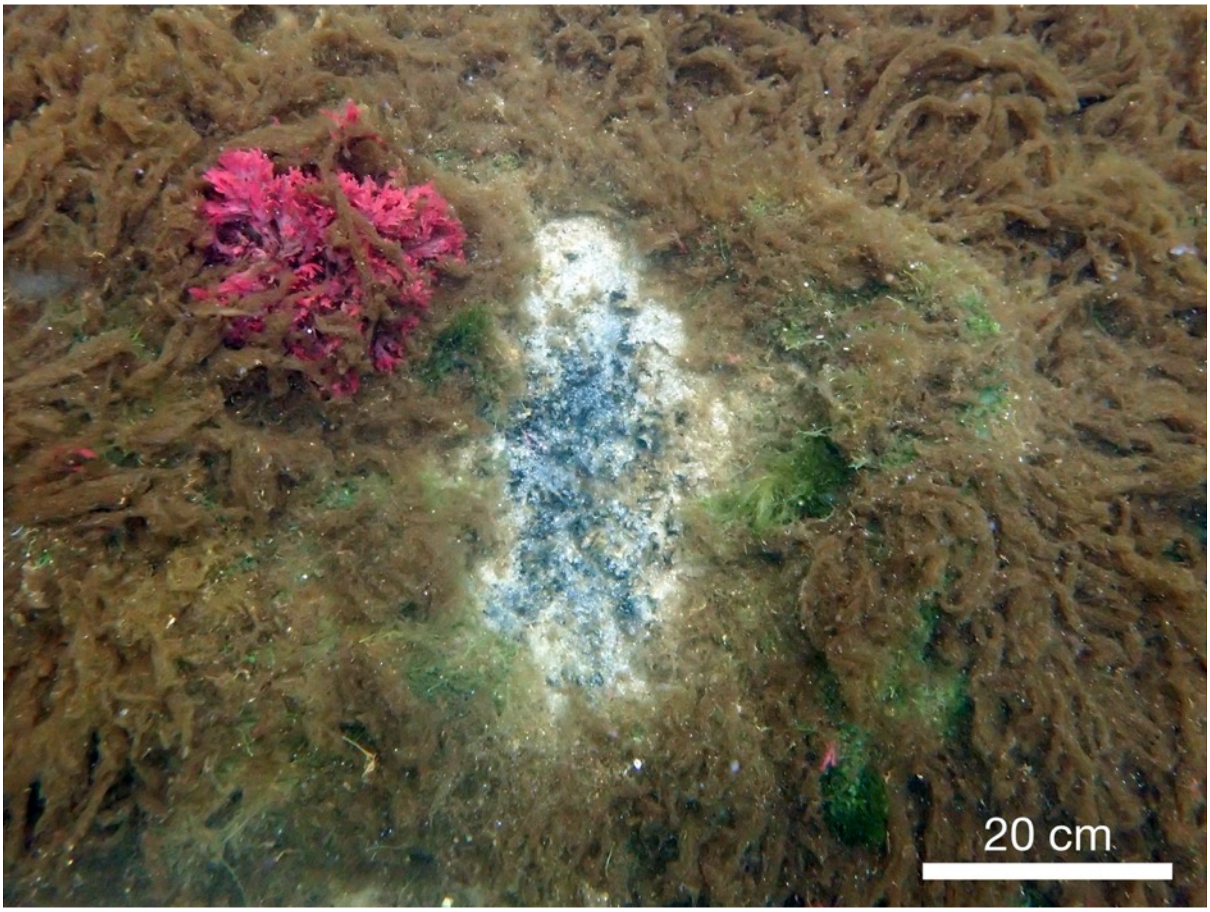

Supplementary Figure 4. Example of the black, anoxic sand located within and underneath the turf algae (after the top layer of turf algae was removed).

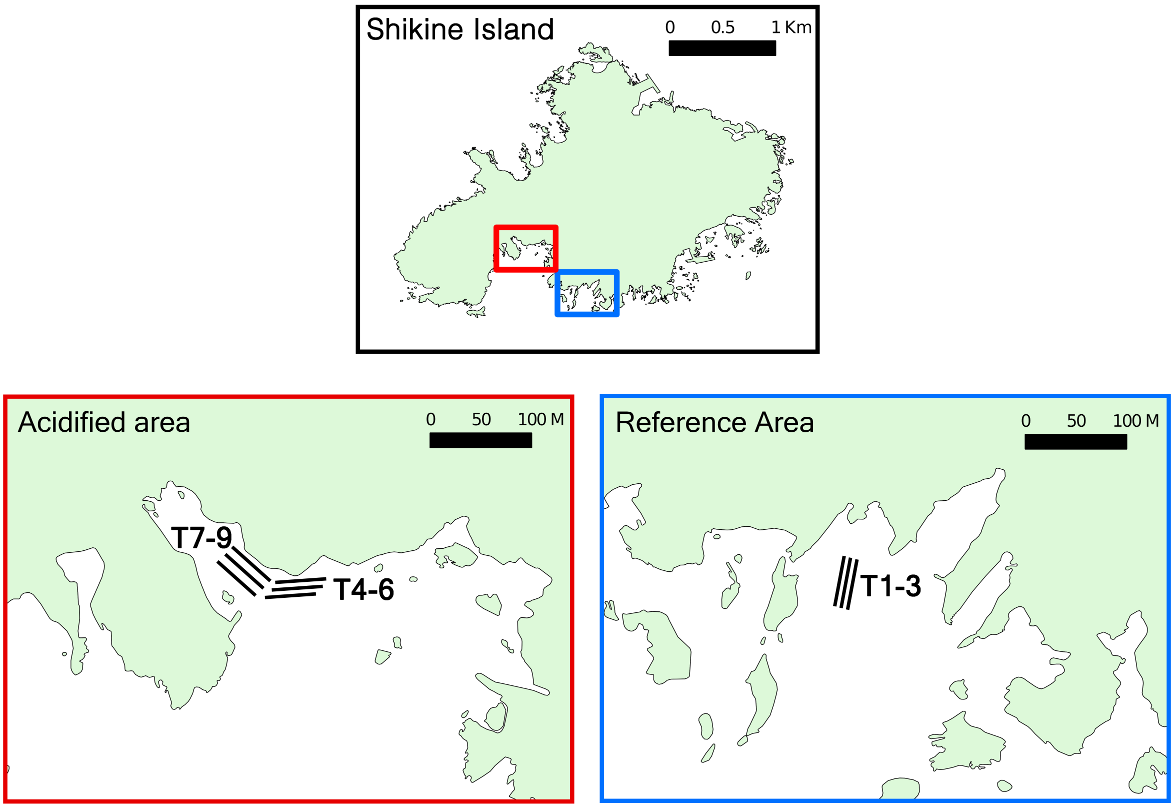

Supplementary Figure 5. Study area showing the nine 50m transect locations (T1-T3) in the reference and (T4-T9) in the acidified area. Transects T4-6 are located between sites 'RCP 2.6' and 'RCP 8.5', and Transects T7-T9 are located between 'RCP 8.5' and '> RCP 8.5'.

Supplementary References

Agostini, S., Harvey, B. P., Wada, S., Kon, K., Milazzo, M., Inaba, K., & Hall-Spencer, J. M. (2018). Ocean acidification drives community shifts towards simplified non-calcified habitats in a subtropical-temperate transition zone. *Scientific Reports*, *8*, 11354. https://doi.org/10.1038/s41598-018-29251-7

Agostini, S., Wada, S., Kon, K., Omori, A., Kohtsuka, H., Fujimura, H., Tsuchiya, Y., Sato, T., Shinagawa, H., Yamada, Y., & Inaba, K. (2015). Geochemistry of two shallow CO_2 seeps in Shikine Island (Japan) and their potential for ocean acidification research. *Regional Studies in Marine Science*, *2, Supplement*, 45–53.

Harvey, B. P., Agostini, S., Kon, K., Wada, S., & Hall-Spencer, J. M. (2019). Diatoms dominate and alter marine food-webs when CO_2 rises. *Diversity*, *11*(12). https://doi.org/10.3390/d11120242

IPCC. (2013). *Climate Change 2013 - The Physical Science Basis: Working Group I Contribution to the Fifth Assessment Report of the IPCC* (No. 0521880092; p. 1535). Cambridge University Press.

Rajaram, S., & Oono, Y. (2010). NeatMap - non-clustering heat map alternatives in R. *BMC Bioinformatics*, *11*(1), 45. https://doi.org/10.1186/1471-2105-11-45
