# Supplementary material for: Feedback mechanisms stabilise degraded turf algal systems at a CO2 seep site
Source: Commun Biol. 2021 Feb 16;4:219. doi: 10.1038/s42003-021-01712-2 (PMC7901039; doi:10.1038/s42003-021-01712-2)
Supplement: Supplementary file 3 — Reporting Summary [file 42003_2021_1712_MOESM3_ESM.pdf]

nature research

Corresponding author(s): Ben P. Harvey
Last updated by author(s): Nov 26, 2020

Reporting Summary

Nature Research wishes to improve the reproducibility of the work that we publish. This form provides structure for consistency and transparency in reporting. For further information on Nature Research policies, see our Editorial Policies and the Editorial Policy Checklist.

Statistics

For all statistical analyses, confirm that the following items are present in the figure legend, table legend, main text, or Methods section.

n/a	Confirmed	
☐	☒	The exact sample size (*n*) for each experimental group/condition, given as a discrete number and unit of measurement
☐	☒	A statement on whether measurements were taken from distinct samples or whether the same sample was measured repeatedly
☐	☒	The statistical test(s) used AND whether they are one- or two-sided<br>*Only common tests should be described solely by name; describe more complex techniques in the Methods section.*
☒	☐	A description of all covariates tested
☐	☒	A description of any assumptions or corrections, such as tests of normality and adjustment for multiple comparisons
☐	☒	A full description of the statistical parameters including central tendency (e.g. means) or other basic estimates (e.g. regression coefficient) AND variation (e.g. standard deviation) or associated estimates of uncertainty (e.g. confidence intervals)
☐	☒	For null hypothesis testing, the test statistic (e.g. *F*, *t*, *r*) with confidence intervals, effect sizes, degrees of freedom and *P* value noted<br>*Give P values as exact values whenever suitable.*
☒	☐	For Bayesian analysis, information on the choice of priors and Markov chain Monte Carlo settings
☒	☐	For hierarchical and complex designs, identification of the appropriate level for tests and full reporting of outcomes
☒	☐	Estimates of effect sizes (e.g. Cohen's *d*, Pearson's *r*), indicating how they were calculated

Our web collection on statistics for biologists contains articles on many of the points above.

Software and code

Policy information about availability of computer code

Data collection	When assessing the 'change in dominant habitat type' and 'differences in the forward and reverse threshold along the pCO2 gradient' - Photoquadrats were analysed using ImageJ (Version: 2.0.0-rc-69/1.51i) by overlaying 64 points on a grid, and recording the presence of turf algae, other macroalgae, or coral.
Data analysis	Data analysis and statistics for each section was carried out using R (Version: 3.6.2), including ANOVA, Kruskal-Wallis, Tukey-HSD post-hoc test, and PERMANOVA. Carbonate chemistry was calculated using CO2SYS.

For manuscripts utilizing custom algorithms or software that are central to the research but not yet described in published literature, software must be made available to editors and reviewers. We strongly encourage code deposition in a community repository (e.g. GitHub). See the Nature Research guidelines for submitting code & software for further information.

Data

Policy information about availability of data

All manuscripts must include a data availability statement. This statement should provide the following information, where applicable:

- Accession codes, unique identifiers, or web links for publicly available datasets
- A list of figures that have associated raw data
- A description of any restrictions on data availability

Raw data used for Figures 3-8 are freely accessible and stored on figshare (DOI: 10.6084/m9.figshare.13289588). Raw DNA sequences used for microbial community analyses are accessible through the European Nucleotide Archive (accession: PRJEB40334).

Field-specific reporting

Please select the one below that is the best fit for your research. If you are not sure, read the appropriate sections before making your selection.

☐ Life sciences ☐ Behavioural & social sciences ☒ Ecological, evolutionary & environmental sciences

For a reference copy of the document with all sections, see nature.com/documents/nr-reporting-summary-flat.pdf

Ecological, evolutionary & environmental sciences study design

All studies must disclose on these points even when the disclosure is negative.

Study description

Ocean acidification has strong potential to substantially increase turf algae, which led us to examine the mechanisms that stabilise turf algal states. Our CO_2 seep studies show that ocean acidification promotes turf algae over corals and macroalgae and then reinforces feedback loops (altered physicochemical environment and microbial community, and an inhibition of recruitment) that facilitate turf algal dominance.

Research sample

(i) Change in dominant habitat type. Percentage cover of the substratum and associated carbonate chemistry were assessed in June 2017 using nine 50 m transects along the CO_2 gradient, with four photoquadrats (50 x 50 cm) and a seawater sample (100 ml) taken every 5 m along the transects.
(ii) Effects of habitat simplification on environmental conditions - Field-based
To assess indirect effects of turf algae on water chemistry, 15 sets of measurements were taken (by syringe) from haphazard locations in turf algae growing in elevated pCO_2 conditions. For each set of measurements, four water samples were collected: 'Sediment', 'Turf Algae', 'Surface', and 'Seawater'.
(iii) Effects of habitat simplification on environmental conditions - Laboratory-based
Turf algal microcosms were set up in the laboratory to measure within habitat pH profiles using microsensors for comparison with field observations. We simulated turf algal habitats (n = 3) by placing a 5 cm layer of sediment collected from underneath turf algae at our study site into a vertical (30 cm H x 10 cm D) clear Perspex tube and overlaying this with a 25 mm thick layer of turf algae (also from the study site).
(iv) Effects of altered habitat on associated microbial communities
To characterise the composition of microbial communities associated with turf algae, samples were collected by hand from: (i) the surface of the turf algae, (ii) the middle of the turf algae, and (iii) the sediment below the turf algae, from eight haphazard locations within the elevated pCO_2 area (24 samples in total, n = 8).
(v) Effects of altered habitat type on recruitment of other species
We attached ten PVC tiles (150 x 150 mm) to sublittoral rock at 6-7 m depth in an elevated pCO_2 area at the end of March 2018 (at the beginning of the spring growth of turf algae). Tiles were separated by at least 3 m horizontal distance and were placed into the same light and wave exposure conditions. The tiles were retrieved in July 2018 and the turf gently removed to photograph any recruits underneath.
(vi) Testing for differences in the forward and reverse threshold along the pCO_2 gradient
We attached plastic green mesh tiles (150 x 150 mm) to the sublittoral rock at 6-7 m depth in the most elevated pCO_2 area (based on the transects used in the 'Change in dominant habitat type' subsection) in March 2019 (at the beginning of the spring growth of turf algae). Tiles were separated by at least 3 m horizontal distance and were placed into the same light and wave exposure conditions. Once the tiles were fully covered by turf algal assemblages in late May 2019, the tiles were then transplanted back along the pCO_2 gradient. The transplanted tiles were attached to the sublittoral rock at 6-7 m at five different locations (Reference pCO_2, pH_{NBS} 8.17 ± 0.001; and four elevated pCO_2 locations, pH_{NBS} 8.04 ± 0.04, 7.91 ± 0.04, 7.87 ±0.02, 7.85 ± 0.02 respectively), with five replicates at each location (25 tiles in total). The percentage cover of the remaining turf algal assemblage was assessed after 1-month (late June) and 2-months (late July) taking a photograph of each tile in-situ (Olympus TG-5 Camera). Analysis was performed using ImageJ by overlaying 64 points on a grid, and recording the presence of turf algae (using the same approach used in the section 'Change in dominant habitat type').

Sampling strategy

As this was a field-based study on a remote island, the sample sizes for each experiment within the study were determined by logistics due to the availability of working hours, safety considerations for diving, boat time and suitable weather conditions. The replication of the molecular analysis of the microbial communities was determined by a budget for sequencing costs.

Data collection

(i) Change in dominant habitat type.
Transects with photoquadrats and water samples were collected in situ by Ben P. Harvey (BH) and Ro Allen (RA). Water samples were immediately measured for carbonate chemistry parameters on RV Tsukuba II by BH. Photographs were analysed using ImageJ by BH.
(ii) Effects of habitat simplification on environmental conditions - Field-based
Water samples were collected in situ and immediately measured for dissolved oxygen and carbonate chemistry parameters on RV Tsukuba II by BH and Shigeki Wada (SW).
(iii) Effects of habitat simplification on environmental conditions - Lab-based
Turf and sediment samples were collected by hand, in situ and returned to the laboratory for microsensor analyses by BH and Sylvain Agostini (SA).
(iv) Effects of altered habitat on associated microbial communities
Samples were collected by hand, in situ by BH and RA. Molecular analysis of the samples was performed by RA, Linn J. Hoffmann (LH) and Tina C. Summerfield (TS).
(v) Effects of altered habitat type on recruitment of other species
Recruitment tiles were deployed, and later collected, by BH and Jason M. Hall-Spencer. Photographs were analysed using ImageJ by BH.
(vi) Testing for differences in the forward and reverse threshold along the pCO_2 gradient
Transplanted turf algal assemblages were deployed and photographed by BH, SW and SA. Photographs were analysed using ImageJ

	by BH.<br>Statistical analysis for experiments (i), (ii), (iii), (v) and (vi) were performed in R by BH. Statistical analysis for experiment (iv) was performed in R by RA, LH and TS.
Timing and spatial scale	Surveys and experiments took place in June 2017, with the 'effects of altered habitat type on recruitment of other species' carried out in March-July 2018, and the 'Testing for differences in the forward and reverse threshold along the pCO2 gradient' performed March-July 2019.<br>In terms of spatial scale, the elevated-CO2 site used within this study conservatively covers more than 400 m-2, and all sampling is randomly collected from within this site in order to provide independence between samples.
Data exclusions	No data was excluded.
Reproducibility	The aim of the experiment was to examine the mechanisms that stabilise turf algal states under elevated CO2 levels. We have assessed the dynamics of the turf algae in the volcanic seep off Shikine Island seasonally through scuba diving from 2015–2020, and have observed the same patterns every year.
Randomization	Samples within the site were randomly chosen. For the transects used in '(i) Change in dominant habitat type', the transects were orientated along known gradients in CO2, however the photoquadrats and water samples taken every 5m along the transect were randomly chosen. For the transplanted turf algae, the sites used for the transplants were chosen along the known gradients in CO2, but the deployment locations within each location were randomly chosen.
Blinding	This research was done underwater at CO2 seeps. Hence, visual navigation to the sites was necessary, but the choice of samples were randomly chosen. For the measurements of the water samples, the person taking the measurements (BH) was blinded to the sample details, and was given the samples in a random order - so that only the scribe knew which sample they were measuring.

Did the study involve field work? ☒ Yes ☐ No

Field work, collection and transport

Field conditions	Shikine Island (34°19′9″ N, 139° 12′18″ E) is part of a group of offshore volcanic islands in Japan, termed the Izu Islands. A gradient of seawater CO2 concentration is present in the area due to a CO2 seep. The elevated pCO2 areas are not confounded by differences in temperature, dissolved oxygen, total alkalinity, nutrients or depth relative to reference sites used for comparison.
Location	We assessed the responses of shallow (0-10 m depth) coral and algal habitats along a gradient of seawater CO2 concentrations at CO2 seeps off Shikine Island, Japan (34°19′9″ N, 139° 12′18″ E). Field experiments and surveys were carried out in the same locations.
Access & import/export	Site access is achieved via boat (RV Tsukuba II, University of Tsukuba) and SCUBA-diving. Permission was obtained through a working relationship with the Shikine Island Fisheries Agency, and a Permit (Tokyo, 2017 - Permit Number: 29-12; Issued; 2017-05-23 to 2018-04-31). All samples were processed within Japan at the Shimoda Marine Research Center, University of Tsukuba.
Disturbance	Disturbance was minimal. Samples of the turf algae were collected for the laboratory (when simulating the turf algal habitats) where in total only around 0.8m-2 area was collected - where the species conservatively covers more than 400 m-2. Samples of the turf algae were also collected for associated microbial communities, however these were collected in 2ml cryovials and therefore required only small samples. Recruitment tiles and transplanted turf assemblages were deployed and then retrieved at the end of the experiment - causing minimal disturbance to the ecosystem.

Reporting for specific materials, systems and methods

We require information from authors about some types of materials, experimental systems and methods used in many studies. Here, indicate whether each material, system or method listed is relevant to your study. If you are not sure if a list item applies to your research, read the appropriate section before selecting a response.

Materials & experimental systems

n/a	Involved in the study
☒	☐ Antibodies
☒	☐ Eukaryotic cell lines
☒	☐ Palaeontology and archaeology
☐	☒ Animals and other organisms
☒	☐ Human research participants
☒	☐ Clinical data
☒	☐ Dual use research of concern

Methods

n/a	Involved in the study
☒	☐ ChIP-seq
☒	☐ Flow cytometry
☒	☐ MRI-based neuroimaging

Animals and other organisms

Policy information about studies involving animals; ARRIVE guidelines recommended for reporting animal research

Laboratory animals	Study did not involve laboratory animals.

Wild animals	Study did not involve wild animals.
Field-collected samples	Study did not involve experiments that used field-collected animal samples.<br><br>Algal samples that were collected from the field were maintained under CO2 controlled running seawater with 12:12 light conditions for one week before the pH profile of the community was measured. Following the experiment, the turf algae was disposed of.
Ethics oversight	No ethical approval or guidance was required as the only experimental organism was algae.

Note that full information on the approval of the study protocol must also be provided in the manuscript.
